# Micro-CT data of complete metamorphosis process in *Harmonia axyridis*

**DOI:** 10.1038/s41597-024-03413-x

**Published:** 2024-05-30

**Authors:** Runguo Shu, Yiqi Xiao, Chaowei Zhang, Ying Liu, Hang Zhou, Fei Li

**Affiliations:** 1 State Key Laboratory of Rice Biology & Ministry of Agricultural and Rural Affairs Key Laboratory of Molecular Biology of Crop Pathogens and Insects, Institute of Insect Sciences, Zhejiang University, Hangzhou, 310058 China; 2 Key Laboratory of Green Prevention and Control of Agricultural Transboundary Pests of Yunnan Province, Agricultural Environment and Resource Research Institute, Yunnan Academy of Agricultural Sciences, Kunming, 650205 China

**Keywords:** Biological metamorphosis, Evolutionary developmental biology

## Abstract

Insect metamorphosis involves significant changes in insect internal structure and is thus a critical focus of entomological research. Investigating the morphological transformation of internal structures is vital to understanding the origins of adult insect organs. Beetles are among the most species-rich groups in insects, but the development and transformation of their internal organs have yet to be systematically documented. In this study, we have acquired a comprehensive dataset that includes 27 detailed whole-body tomographic image sets of *Harmonia axyridis*, spanning from the prepupal to the pupal stages. Utilizing this data, we have created intricate 3D models of key internal organs, encompassing the brain, ventral nerve cord, digestive and excretion systems, as well as the body wall muscles. These data documented the transformation process of these critical organs and correlations between the origin of adult and larval organs and can be used to enhance the understanding of holometabolous adult organ genesis and offers a valuable reference model for investigating complete metamorphosis in insects.

## Background & Summary

Insect metamorphosis is the transformation process from the juvenile to the adult stage, playing a vital role in the insect lifecycle and encompassing extensive physical and biological changes. Holometabolous insects undergo complete metamorphosis and are characterized by a distinct pupal stage that exhibits profound internal structural transformations^1^. This metamorphosis separates these from other insect groups, clearly delineating life stages and facilitating complex adaptations to various ecological niches^2–4^. The group includes over 85% of insect species, which attests to the evolutionary success of the complete metamorphosis^5^. The investigation of the internal transformations associated with metamorphosis is a crucial focus of entomological studies, providing insights into the life cycle and developmental origin of this process.

The non-transparent nature of the body wall in many holometabolous insects makes it challenging to observe changes in their internal structures during development. To overcome this, various techniques were employed, including light microscopy, scanning electron microscope (SEM), confocal laser microscopy, and micro-computed tomography (micro-CT)^6^. Each method has specific benefits and limitations, such as the requirement for semi-transparent or removable body walls for light microscopy, and size constraints for SEM^7,8^. In addition, confocal laser microscopy requires fluorescent labelling and slicing of the tissue^9,10^. The miniaturization of X-ray imaging equipment has helped circumvent this problem, leading to the widespread application of micro-CT in studying the metamorphosis stages of insects across various orders. Previous studies provided important details on the prepupal stage of *Calliphora vicina* and *Chrysopa pallens*^11,12^, while others mainly focused on changes occurring during the pupal stage^13–16^. Notably, the prepupal stage, which marks the beginning of complete metamorphosis and is characterized by significant morphological changes, has remained understudied.

Phylogenetic analysis shows that the Coleoptera differentiated more than 280 million years ago, a split that predates other major holometabolous insect orders^17^. The larvae of some Coleopteran insects, particularly within the Coccinellidae, bear a closer resemblance to the wingless nymphs of hemimetabolous insects. This similarity reflects an intermediate evolutionary stage that makes Coleoptera insects particularly suited for research into the developmental origin of adult organs. However, the development and transformation of their internal organs have yet to be systematically documented.

The harlequin ladybird, *Harmonia axyridis*, is a native Asian insect and a significant biological control agent that is considered an invasive species globally^18,19^, serving as a model for research in invasion biology^20^. In recent years, the potential of this insect group as a model organism for genetic and developmental studies has also been recognized^21–23^, making them an excellent material for studying the internal structural changes of Coleoptera insects during metamorphosis process.

**Fig. 1:**
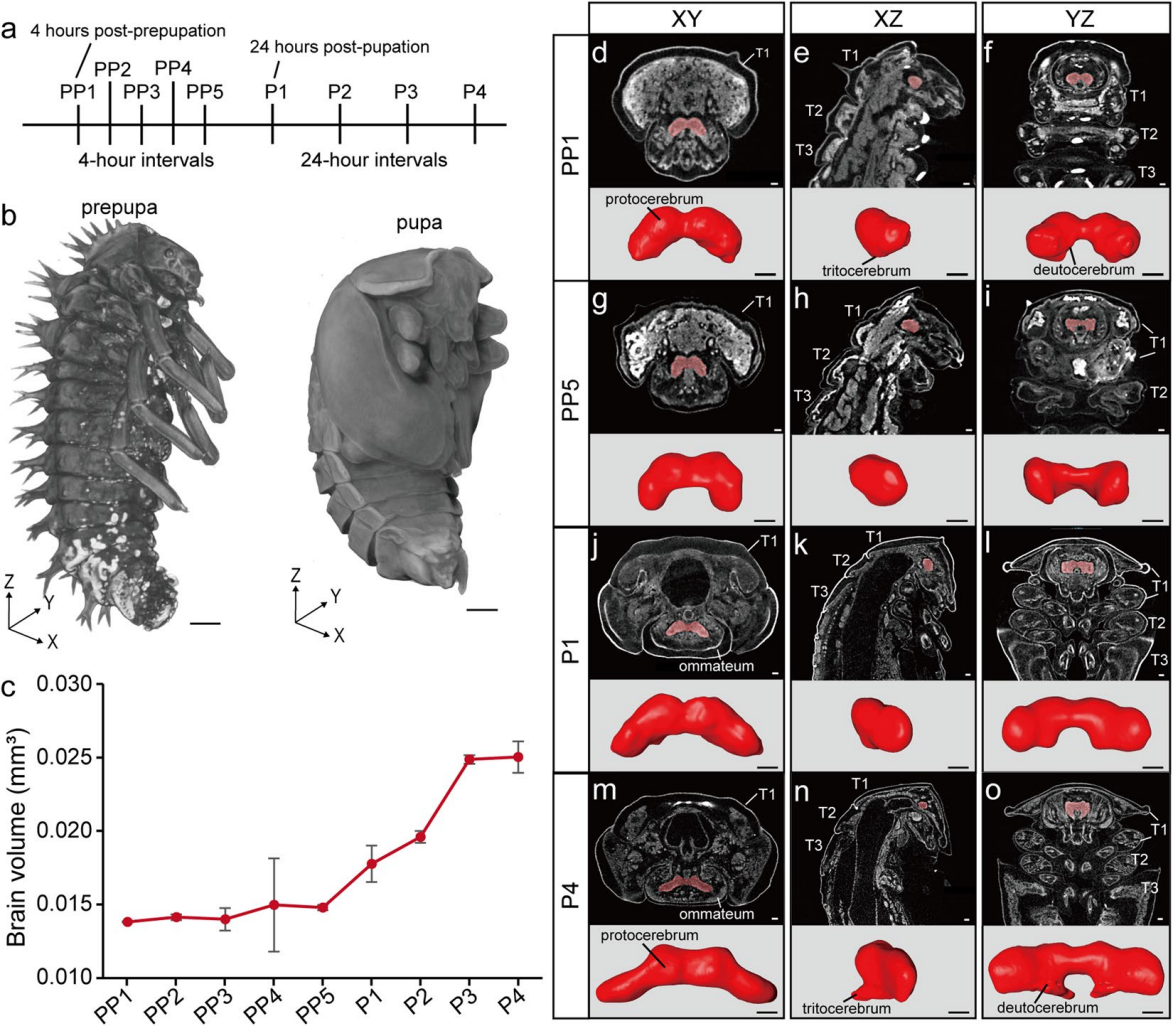
*Harmonia axyridis* 3D reconstructions of the brain, from prepupa to late pupa. (**a**) Diagram of sampling time points. (**b**) 3D View of prepupa and pupa. (**c**) Quantification of brain volume during metamorphosis. The error bars indicate the standard error for three specimens at each stage. (**d**–**o**) Dorsal, lateral, ventral view of the brain at different time points. The brain is marked in red in the different images. T: thoracic segment. Scale bar: 0.1 mm.

The harlequin ladybird prepupal stage begins in the late phase of the fourth instar larva. As detailed in the Methods section, we started sampling every 4 hours from individuals entering the prepupal stage, which changed to 24 hours upon the start of the pupal stage (Fig. 1a). This includes sampling of a total of five prepupal stages and four pupal stages, which were selected for micro-CT scanning and 3D modeling (Table 1). Due to precision limitations, micro-CT is not able to delineate the organs of small insects in great detail. Hence, the primary focus of this study was the continuous changes in internal structures, including the brain, ventral nerve cord, digestive system, excretion system, and body wall muscles.

**Table 1:** Overview of datasets.

Label	Age (hours)	Images	Size	Format
PP1-01	4	960	939 MB	16-bit tiff
PP1-02		1042	996 MB	16-bit tiff
PP1-03		1010	1.25 GB	16-bit tiff
PP2-01	8	1060	903 MB	16-bit tiff
PP2-02		1055	898 MB	16-bit tiff
PP2-03		1047	1.29 GB	16-bit tiff
PP3-01	12	1058	901 MB	16-bit tiff
PP3-02		1054	1.00 GB	16-bit tiff
PP3-03		1047	1.18 GB	16-bit tiff
PP4-01	16	1042	996 MB	16-bit tiff
PP4-02		1041	995 MB	16-bit tiff
PP4-03		1062	1.42 GB	16-bit tiff
PP5-01	20	1040	994 MB	16-bit tiff
PP5-02		1034	998 MB	16-bit tiff
PP5-03		1042	996 MB	16-bit tiff
P1-01	48	1038	1.17 GB	16-bit tiff
P1-02		1056	899 MB	16-bit tiff
P1-03		1050	1.19 GB	16-bit tiff
P2-01	72	1038	1.17 GB	16-bit tiff
P2-02		1051	1.14 GB	16-bit tiff
P2-03		1048	1.30 GB	16-bit tiff
P3-01	96	962	1.04 GB	16-bit tiff
P3-02		1041	1.78 GB	16-bit tiff
P3-03		1048	1.30 GB	16-bit tiff
P4-01	120	1046	1.45 GB	16-bit tiff
P4-02		1046	1.45 GB	16-bit tiff
P4-03		1046	1.45 GB	16-bit tiff

PP refer to prepupa, P refer to pupa. The age of each individual is measured commencing from the onset of the prepupal stage.

The insect central nervous system comprises two primary components: the brain and the ventral nerve cord. In harlequin ladybirds, brain volume gradually increases during the prepupal stages (PP1-PP5), followed by a rapid expansion from late prepupal (PP5) to the first day of pupa (Fig. 1c). Maximum volume is achieved on the third day of the pupal stage (P3), after which growth plateaus (Fig. 1c). At PP1, a dorsal view of the brain revealed kidney-shaped lobes with partial fusion of the protocerebrum (Fig. 1e), while lateral views showed a small, downward-protruding tritocerebrum (Fig. 1f). By PP5, the lobes elongated towards the mouthparts, paralleling each other (Fig. 1g). Subsequently, on the first day of the pupal stage (P1), they expanded to both sides (Fig. 1j). A ventral perspective showed an arch-shaped connection between the lobes (Fig. 1l), which continued to extend to both sides and connected with the nerves of the optic lobes (Fig. 1m). At the same time, the tritocerebrum enlarged and developed a noticeable protrusion towards the thoracic segments (Fig. 1n).

**Fig. 2:**
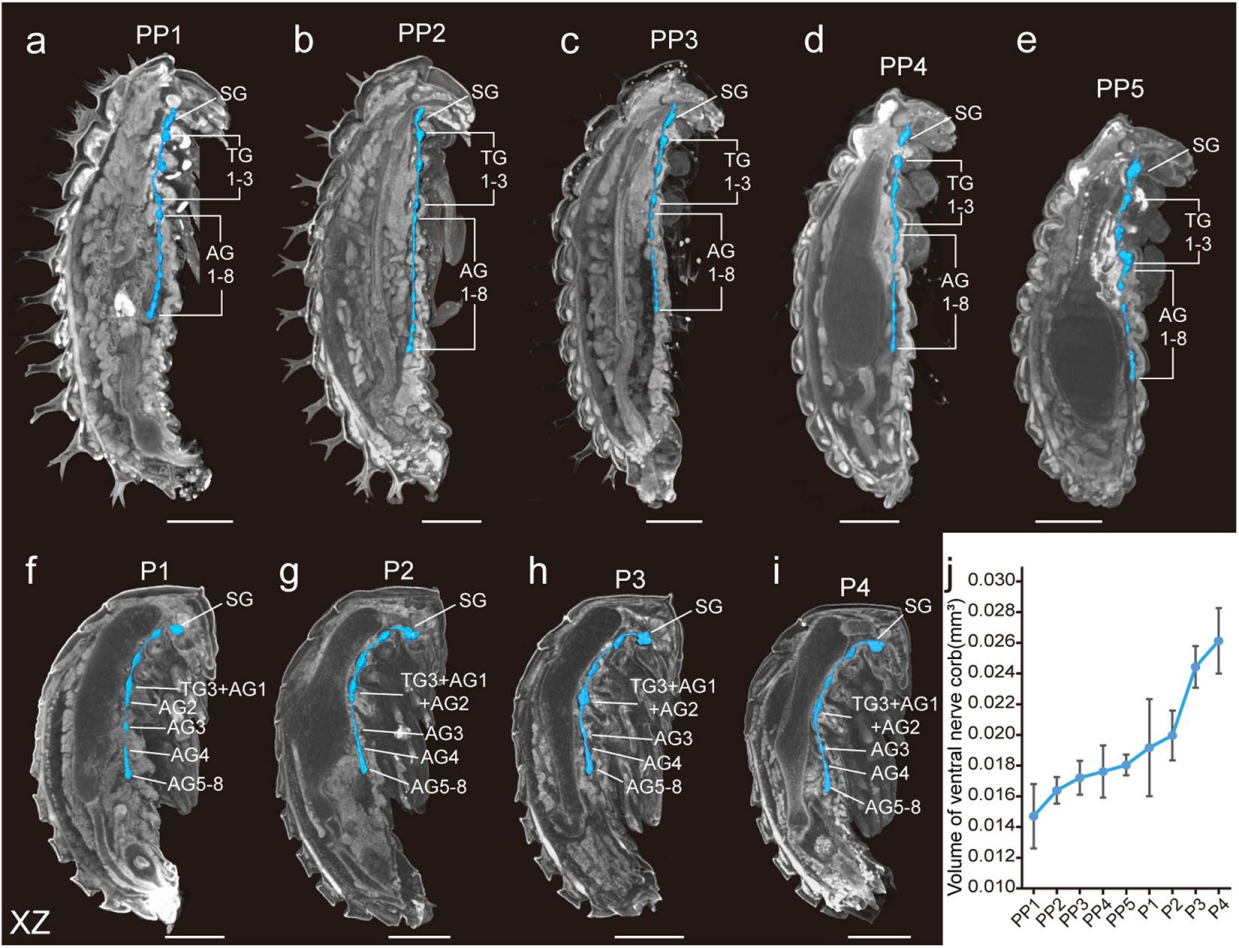
Developmental process of the ventral nerve cords in *Harmonia axyridis* during metamorphosis. (**a**-**i**) Lateral section view of *H. axyridis*, from prepupa to late pupa. The ventral nerve cords are marked in light blue. SG: suboesophageal ganglion, TG: thoracic ganglion, AG: abdomen ganglion. Scale bar: 0.5 mm. (**j**) Quantification of the ventral nerve cord volume during metamorphosis. The error bars indicate the standard error for three specimens at each stage.

At PP1, the larva contained a total of 12 ganglia in the ventral nerve cord, including one suboesophageal ganglion (SG), three thoracic ganglia (TG), and eight abdominal ganglia (AG), as observed from the lateral perspective (Fig. 2). At PP5, the third TG and the first AG began to merge (Fig. 2e) and completely fused into a larger one, with the second AG also integrating with it (Fig. 2f). Eventually, the last three AGs fully merged, resulting in only seven distinct ganglia (Fig. 2i). From PP1 to P5, and despite the decrease in ganglia, the volume of the ventral nerve cord increased (Fig. 2j).

**Fig. 3:**
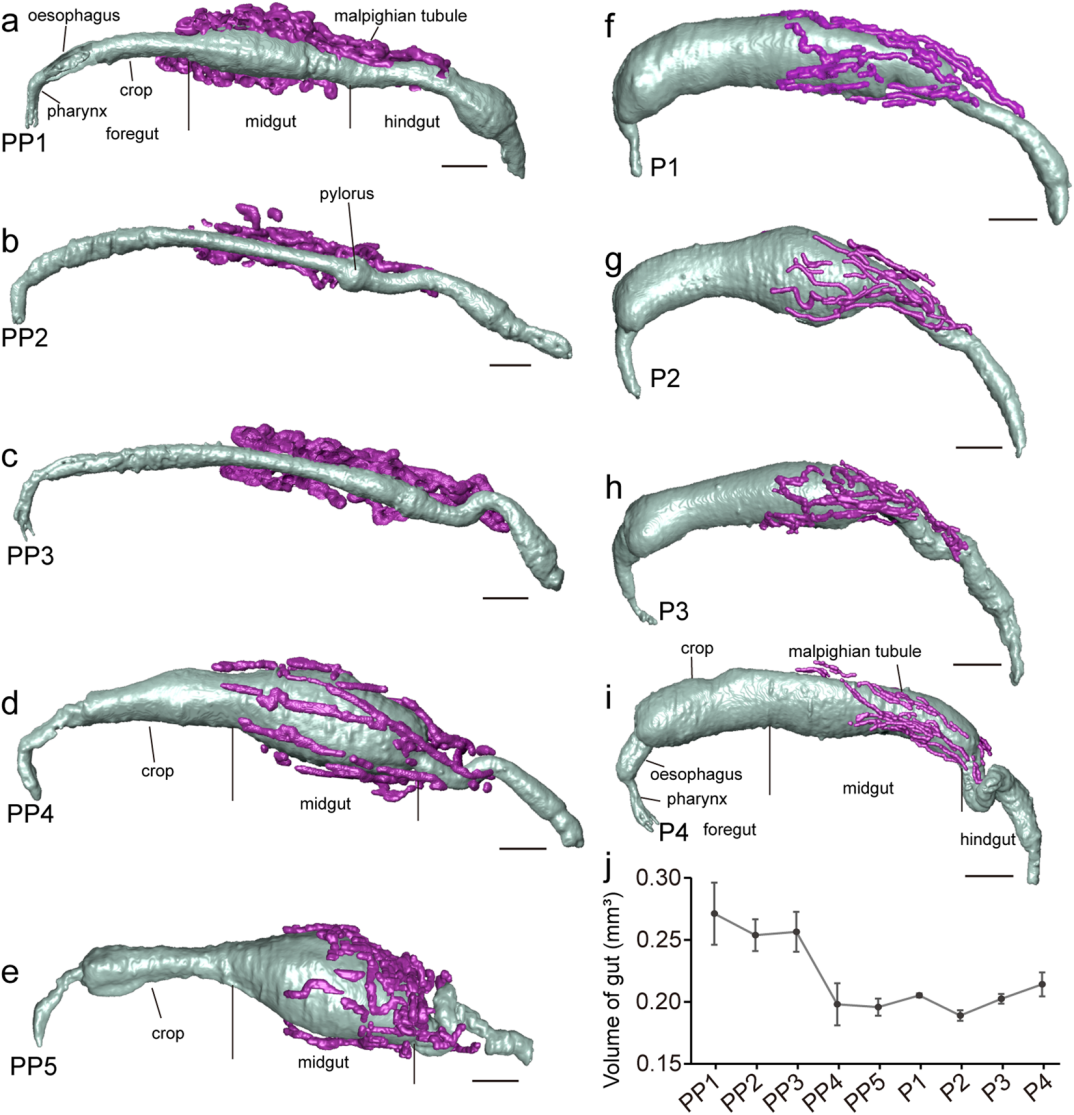
The transformation process of intestinal tracts and Malpighian tubules in *Harmonia axyridis*. (**a**–**i**) Lateral view of intestinal tracts and Malpighian tubules, from prepupa to pupa. The intestinal tracts are marked in grey, and the Malpighian tubules are marked in purple. Scale bar: 0.2 mm. (**j**) Quantification of the intestinal volume during metamorphosis. The error bars indicate the standard error for three specimens at each stage.

At PP1, the crop in the foregut was contracted, whereas the midgut appeared swollen (Fig. 3a). The anterior section contracted in the hindgut, and the posterior section bulged. The Malpighian tubules are highly coiled at this stage, extending from the anterior part of the hindgut to the midgut, fully covering the latter (Fig. 3a). Four hours later, the midgut had contracted, revealing a prominent bulge at its junction with the hindgut, which constituted the pylorus (Fig. 3b). During the transition from PP4 to PP5, the midgut underwent marked swelling, creating an internal cavity, and the crop also enlarged (Figs. 2d,e, 3d,e). Concurrently, the hindgut became coiled, while the Malpighian tubules reduced and could not fully envelope the midgut (Fig. 3d,e).

Upon entering the pupal stage, the crop fully expanded, the midgut swelling reduced, and the hindgut was uncoiled (Fig. 3f). From P1 to P5, the Malpighian tubules gradually disintegrated, eventually covering only the latter half of the midgut and becoming noticeably thinner, while the hindgut recoiled (Fig. 3f–i). The intestinal volume decreased from the PP1 to PP4 stages, then stabilized in size, with a slight increase before eclosion occurred (Fig. 3j).

**Fig. 4:**
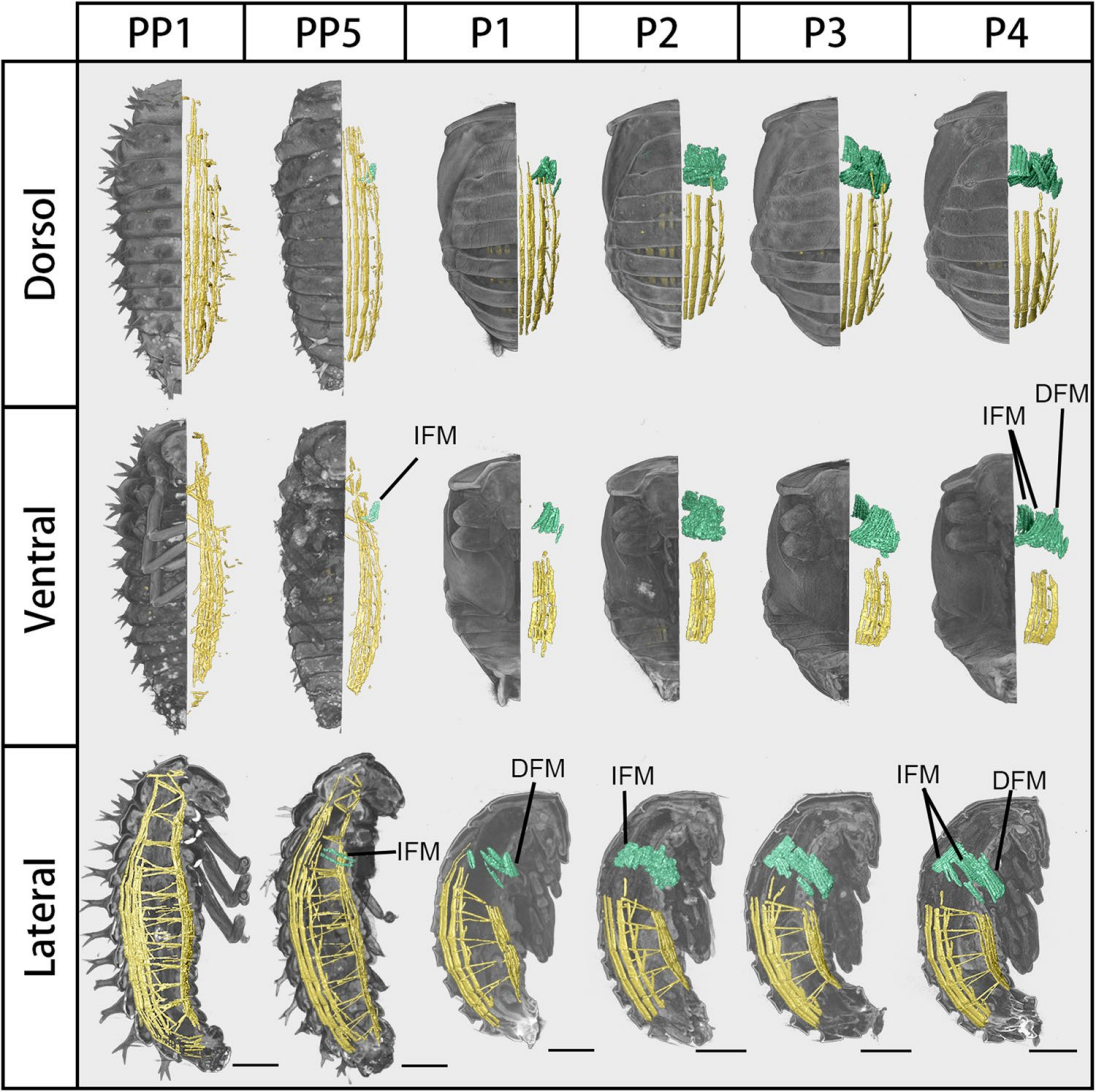
Developmental process of body wall muscles in *Harmonia axyridis* during metamorphosis. Dorsal, ventral, and lateral views of body wall muscles, from prepupa to pupa. The peripheral body wall muscles are marked in yellow, while the flight muscles are marked in green. IFM: indirect flight muscle, DFM: direct flight muscle. Scale bar: 0.5 mm.

Due to the impossibility of visually and computationally distinguishing muscle grayscale values in other appendages, we primarily focused on changes occurring in the body wall muscles of the trunk and flight muscles, as shown in Fig. 4. The prepupa possesses four dorsal longitudinal muscles on the right side, extending from the abdomen to the thorax, as evident in the dorsal view. Owing to bilateral symmetry, there are eight dorsal longitudinal muscles in total. The ventral side possesses an equal number of longitudinal muscles as the dorsal side, with those nearer the midline being longer. Laterally, each body segment was interspersed with small muscle bundles, linking the external muscles of both the dorsal and ventral regions. From PP5 to P1, the thoracic body wall muscles rapidly degraded. By the late pupal stage P4, all body wall muscles in the thorax, except for the flight muscles, were virtually unobservable.

The indirect flight muscles (IFM) first emerged in the late prepupal stage (PP5) and are oriented perpendicularly to the dorsal plate. At P1, the direct flight muscles (DFM) appeared close to the lateral body wall. The IFMs, aligned parallel to the dorsal plate, become visible only on P2. The flight muscles compact in the day preceding eclosion, rendering each muscle bundle distinctly visible.

In summary, we employed micro-CT analysis to model and illustrate major internal structural changes during the metamorphic development of the harlequin ladybird, encompassing the transition from prepupal to late pupal stages. To our knowledge, this study presents the first 3D internal structural atlas for Coleoptera insects. The transformation processes of various organs were systematically described. The origins and time of emergence of various adult organs were determined. This extensive research provides an invaluable resource, significantly enhancing the understanding of insect metamorphosis and offering a helpful reference data for investigating complete metamorphosis in insects.

## Methods

Adult ladybirds were reared in mesh cages measuring 30 × 30 × 30 cm, while the larvae used for the experiments were kept in transparent plastic boxes measuring 20 × 10 × 8 cm and equipped with air holes. The aphids used for feeding the ladybirds were raised on broad bean plants, which were cut and placed into containers kept at 25 ± 1 °C and 75% relative humidity. To accurately determine the stages of prepupa, we employed a meticulous observation method. Larvae were continuously monitored using a Sony FDR-AX60 video camera, allowing us to document their behaviour and developmental changes. Specifically, the onset of the prepupa stage was defined when larvae ceased movement and exhibited a curled posture, marked as 0 hour, after which we took samples every 4 hours. Subsequent samplings were timed from this point onwards. Entry into the pupal stage was identified by the shedding of the exoskeleton, marked as 0 day, with further samples taken based on this timing. Sampling occurred every 24 hours (Fig. 1a).

The insects were anesthetized with carbon dioxide and transferred to a 75% ethanol solution for one day. Every specimen was immediately deceased, ensuring that their developmental stage at the time of sampling remained unchanged throughout the scanning process. Subsequently, the samples were washed three times with 1 mL of phosphate-buffered saline (PBS) and stained with 1 mL of standard Lugol’s solution for one week. After this, the samples were washed three times in PBS to remove excess Lugol’s solution and stored in PBS at 25 °C until scanning was performed within one week to obtain accurate morphological and quantitative analyses.

Sample holders consisted of 200 μL pipette tips. Once sealed at high temperatures, these tips were trimmed to a suitable length. PBS was then transferred into the pipette tips. To ensure sample stability, a brush was employed to gently press the sample against the inner wall of the pipette tip until it was immobile. To inhibit the evaporation of the liquid during prolonged scanning procedures, the tip’s opening was sealed with parafilm. In the final assembly, plasticine was used to fix the sample holder to the mount, aligning the pipette tip longitudinally with the mount to minimize any movement during rotational scanning.

The sample was scanned using a Skyscan 1272 desktop high-resolution 3D X-ray microscope. This microscope was operated via a Dell desktop workstation equipped with an Intel Xeon Gold 6128 Processor, 128 GB of memory, and an NVIDIA Quadro P4000 graphics card. The SKYSCAN 1272 software facilitated instrument control, measurement planning, and data acquisition. The X-ray source settings were adjusted to a voltage of 45 kV and a current of 35 μA. X-ray detection was performed by a 16 MP sCMOS detector, which converted them into photons. The scanning resolution was optimized to its maximum, ensuring the entire sample was captured. To mitigate image blurring resulting from sample rotation, random movement correction was set to 30, while frame averaging was adjusted to 6 to enhance the signal-to-noise ratio of the images. In our study, based on the log files generated during the scanning process by the Micro CT, the exposure time for acquiring CT images was set at 480 milliseconds. The total duration for scanning each sample, both during the pupa and prepupa stages, was approximately 45 minutes. Tomographic image generation was performed using the NRecon software (Bruker, v1.7.4).

## Data Records

The reconstructed datasets are publicly accessible on Figshare^24^ (10.6084/m9.figshare.25801615) and InsectBase v2.0^25^ (http://v2.insect-genome.com/Micro-CT). We encourage researchers to utilize either Amira (v5.4.0, Thermo Fisher Scientific, Berlin, Germany) or Dragonfly (v2022.2, Object Research System, Canada) software for the processing of these data, as a reference for insect metamorphosis research.

The reconstructed dataset consists of 27 whole body tomographic image sets of *H. axyridis*, from prepupa to pupa. The age of each individual is measured commencing from the onset of the prepupal stag. See Table 1 for an overview of the data files and their formats. The volumes of various organs can be obtained from Table 2.

**Table 2:** Volumes of *Harmonia axyridis* organs.

Stage	brain (μm^3^)	ventral nerve cord (μm^3^)	digestive system (μm^3^)
**PP1(4 h)**	13878272.00	11852288.00	323394560.00
	13695318.71	12434579.39	272872407.62
	13704192.00	19815424.00	217164288.00
**PP2(8 h)**	13772672.48	14495791.42	236264005.18
	13900321.12	18155371.04	239753357.93
	14551552.00	16513344.00	285144576.00
**PP3(12 h)**	15803392.00	19683840.00	295512064.00
	13037400.27	15050043.79	240692261.56
	12952982.49	16914784.61	233167411.24
**PP4(16 h)**	22378262.44	18365672.38	237572235.15
	13095627.12	13699454.76	189189703.62
	9273945.63	20784960.00	167174322.99
**PP5(20 h)**	14759685.18	16965369.89	212586642.56
	14334976.00	19658752.00	188979712.00
	15111729.17	17506426.45	185445644.13
**P1(48 h)**	20489843.46	24851772.15	202338408.35
	15242381.40	11791402.55	204275997.34
	17414540.12	20867411.24	208712550.82
**P2(72 h)**	18855364.16	18678292.71	214416711.40
	19277777.57	17365080.16	196269607.97
	20517476.85	23852220.39	205983878.63
**P3(96 h)**	24684015.84	23278821.67	199466923.38
	25527650.07	22327542.09	196034069.08
	24312408.52	27714992.00	211775767.51
**P4(120 h)**	23531742.46	21655140.03	203309522.57
	27637039.05	30716011.50	201127770.39
	23844232.20	26024505.44	237676905.20

PP refer to prepupa, P refer to pupa.

## Technical Validation

The quality of the reconstruction is inherently dependent on the characteristics of the original images, with factors such as brightness and contrast playing a pivotal role in shaping the outcome. Due to the inherent variability across samples, reconstruction settings varied slightly; however, standard settings were kept constant, typically encompassing image registration, ring artifact correction, and beam-hardening correction. These parameters can be customized using the NRecon software. During the reconstruction process, we employed the default settings for image registration, along with a ring artifact correction range of 5–10 and a beam-hardening correction set at 95%.

Micro-CT imaging and statistical analyses were conducted using the Dragonfly software (Object Research Systems, v2022.2.0.1361), operated on a Dell workstation equipped with an i9-11900 Intel Core processor, 128 GB of memory, and an Nvidia GeForce RTX 3070Ti graphics card. For segmenting structures into individual Regions of Interest (ROIs), manual delineation was performed using a 2D paintbrush tool. The Otsu algorithm was utilized to efficiently extract high-density areas to minimize human bias in the segmentation process. This algorithm delineates high-density regions by analyzing the grayscale distribution within the selected brush area. Upon completion of ROI delineation, the software directly calculates the corresponding tissue volumes. These ROIs were then transformed into triangular mesh models for enhanced visualization.

## Data Availability

There was no original code in this work.
